# Reproducibility of assessment of full‐dilatation Cesarean section scar in women undergoing second‐trimester screening for preterm birth

**DOI:** 10.1002/uog.26027

**Published:** 2022-09-01

**Authors:** A. Banerjee, Z. Al‐Dabbach, F. E. Bredaki, D. Casagrandi, A. Tetteh, N. Greenwold, M. Ivan, D. Jurkovic, A. L. David, R. Napolitano

**Affiliations:** ^1^ Fetal Medicine Unit, Elizabeth Garrett Anderson Wing University College London Hospital London UK; ^2^ Elizabeth Garrett Anderson Institute for Women's Health, University College London London UK; ^3^ Department of Gynaecology Elizabeth Garrett Anderson Wing, University College London Hospital London UK; ^4^ National Institute for Health Research, University College London Hospitals Biomedical Research Centre London UK

**Keywords:** full‐dilatation Cesarean scar, preterm birth, reproducibility, ultrasound

## Abstract

**Objective:**

To assess the reproducibility of a standardized method of measuring the Cesarean section (CS) scar, CS scar niche and their position relative to the internal os of the uterine cervix by transvaginal ultrasound in pregnant women with a previous full‐dilatation CS.

**Methods:**

This was a prospective, single‐center reproducibility study on women with a singleton pregnancy and a previous full‐dilatation CS who underwent transvaginal ultrasound assessment of cervical length and CS scar characteristics at 14–24 weeks' gestation. The CS scar was identified as a hypoechogenic linear discontinuity of the myometrium at the anterior wall of the lower uterine segment or cervix. The CS scar niche was identified as an indentation at the site of the scar with a depth of at least 2 mm. The CS scar position was evaluated by measuring the distance to the internal cervical os. CS scar niche parameters, including its length, depth, width, and residual and adjacent myometrial thickness, were assessed in the sagittal and transverse planes. Qualitative reproducibility was assessed by agreement regarding visibility of the CS scar and niche. Quantitative reproducibility of CS scar measurements was assessed using three sets of images: (1) real‐time two‐dimensional (2D) images (real‐time acquisition and caliper placement on 2D images by two operators), (2) offline 2D still images (offline caliper placement by two operators on stored 2D images acquired by one operator) and (3) three‐dimensional (3D) volume images (volume manipulation and caliper placement on 2D images extracted by two operators). Agreement on CS scar visibility and the presence of a niche was analyzed using kappa coefficients. Intraobserver and interobserver reproducibility of quantitative measurements was assessed using Bland–Altman plots.

**Results:**

To achieve the desired statistical power, 72 women were recruited. The CS scar was visualized in > 80% of images. Interobserver agreement for scar visualization and presence of a niche in real‐time 2D images was excellent (kappa coefficients of 0.84 and 0.85, respectively). Overall, reproducibility was higher for real‐time 2D and offline 2D still images than for 3D volume images. The 95% limits of agreement (LOA) for intraobserver reproducibility were between ± 1.1 and ± 3.6 mm for all sets of images; the 95% LOA for interobserver reproducibility were between ± 2.0 and ± 6.3 mm. Measurement of the distance from the CS scar to the internal cervical os was the most reproducible 2D measurement (intraobserver and interobserver 95% LOA within ± 1.6 and ± 2.7 mm, respectively). Overall, niche measurements were the least reproducible measurements (intraobserver 95% LOA between ± 1.6 and ± 3.6 mm; interobserver 95% LOA between ± 3.1 and ± 6.3 mm). There was no consistent difference between measurements obtained by reacquisition of 2D images (planes obtained twice and caliper placed), caliper placement on 2D stored images or volume manipulation (planes obtained twice and caliper placed).

**Conclusions:**

The CS scar position and scar niche in pregnant women with a previous full‐dilatation CS can be assessed in the second trimester of a subsequent pregnancy using either 2D or 3D volume ultrasound imaging with a high level of reproducibility. Overall, the most reproducible CS scar parameter is the distance from the CS scar to the internal cervical os. The method proposed in this study should enable clinicians to assess the CS scar reliably and may help predict pregnancy outcome. © 2022 The Authors. *Ultrasound in Obstetrics & Gynecology* published by John Wiley & Sons Ltd on behalf of International Society of Ultrasound in Obstetrics and Gynecology.

**CONTRIBUTION**

*What are the novel findings of this work?*

This study demonstrates that, in women with a previous full‐dilatation Cesarean section (CS), CS scar niche dimensions and scar position relative to the internal os of the uterine cervix can be assessed with a high level of reproducibility using B‐mode and color Doppler transvaginal ultrasound during the second trimester of pregnancy.

*What are the clinical implications of this work?*

Full‐dilatation CS increases the risk of spontaneous preterm birth in a subsequent pregnancy. There are currently no guidelines on how to measure CS scar position in pregnancy. The protocol described in this study should enable objective analysis of the effect of CS scar position and niche during pregnancy, and may be used in future studies to evaluate the associated risk of subsequent preterm birth.

## INTRODUCTION

Globally, the estimated number of births by Cesarean section (CS) almost doubled from 16 million (12%) in 2000 to 29.1 million (21%) in 2015^1^. Furthermore, during the same period, several studies have also documented a concerning increase in the rate of CS at full dilatation (cervical dilatation of 10 cm)^2^, ^3^, ^4^, ^5^. Emerging evidence from recent studies has shown a significant association between full‐dilatation CS and spontaneous preterm birth in subsequent pregnancies^6^, ^7^, ^8^, ^9^, ^10^, ^11^. Levine *et al*. reported a 6‐fold increased risk of spontaneous preterm birth in a subsequent pregnancy following full‐dilatation CS at term compared with first‐stage CS^10^. A reason for this observed association has yet to be determined.

It is hypothesized that a low uterine incision or cervical incision and intraoperative complications, such as tears and extensions of the initial CS incision, may lead to structural damage of the cervix and compromise its function in a subsequent pregnancy^10^, ^12^. Two studies have assessed the visibility and reproducibility of sonographic assessment of the CS scar in pregnancy^13^, ^14^. Naji *et al*. reported that the CS scar was visible in 88.8% of women in the second trimester of pregnancy (19–21 weeks' gestation), with 100% interobserver agreement^13^. The authors also demonstrated good reproducibility of assessment of CS scar characteristics, such as the presence of a niche and scar size, using transvaginal ultrasound in three dimensions. However, these studies did not evaluate the location of the CS scar in relation to the cervix, and were not performed in a cohort of women with a previous full‐dilatation CS. The CS scar characteristics and its position in relation to the internal cervical os after full‐dilatation CS may influence the risk of spontaneous preterm birth in a subsequent pregnancy. A feasibility and reproducibility study is needed to identify parameters that reliably predict pregnancy outcome. The objective of this study was to explore the feasibility and reproducibility of a standardized ultrasound protocol for evaluation of the CS scar and CS scar niche in pregnancy and their position relative to the cervix in women with a previous full‐dilatation CS.

## METHODS

This was a prospective cohort study recruiting women with a singleton pregnancy and a previous full‐dilatation CS who were referred to a dedicated preterm birth surveillance clinic at University College London Hospital, London, UK, between 2019 and 2020. Only women with a confirmed previous lower‐segment full‐dilatation CS were included. Ultrasound assessment of cervical length and CS scar characteristics was performed in the second trimester of pregnancy between 14 and 24 weeks' gestation. Ultrasound imaging was performed using a Voluson E8 Expert ultrasound system (GE Healthcare, Zipf, Austria) with a 4–9‐MHz transvaginal three‐dimensional (3D) probe, without any contrast enhancement with saline or gel. All reproducibility assessments were performed in pairs by seven senior clinicians of the preterm birth clinic team (A.B., A.L.D., Z.A., D.C., A.T., N.G. and R.N.) who were trained in cervical length measurement and underwent a standardization session on CS scar assessment. All operators were blinded to their own and each other's findings, covering the values in mm of measurements on the screen and removing calipers placed on stored images. The detailed ultrasound protocol for CS scar assessment is described in Appendix S1 and Videoclip S1.

The CS scar was defined as a hypoechogenic (or rarely hyperechogenic) discontinuity of the myometrium at the anterior wall of the lower uterine segment or cervix. A niche was defined as an indentation at the site of the CS scar with a depth of at least 2 mm. The CS scar and niche were classified overall as visible, not visible or unclear. A preliminary feasibility study of image acquisition and caliper placement was undertaken in 10 patients. As a result of this feasibility study, six CS scar measurements were considered to be the most relevant and achievable in the second trimester of pregnancy. This was also in keeping with recommendations from previous studies assessing CS scars^15^, ^16^. In addition, cervical length measurements were acquired.

**Figure 1:**
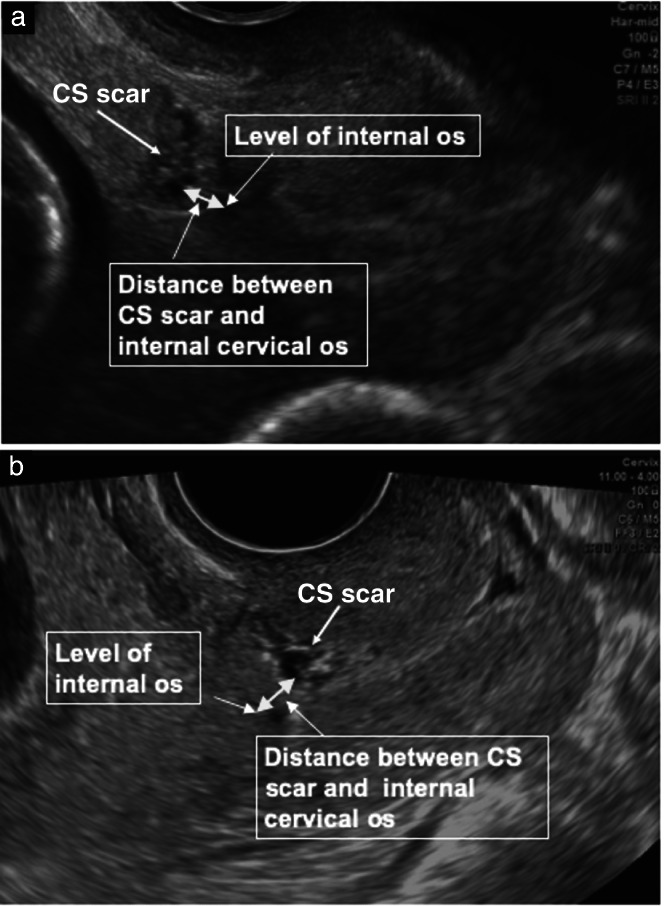
Grayscale ultrasound images showing measurement of the distance from the Cesarean section (CS) scar to the internal cervical os in the sagittal plane in a case with a CS scar above the cervix (a) and a case with a CS scar in the cervix (b). The level of the internal os was determined using color flow mapping of the uterine artery.

**Figure 2:**
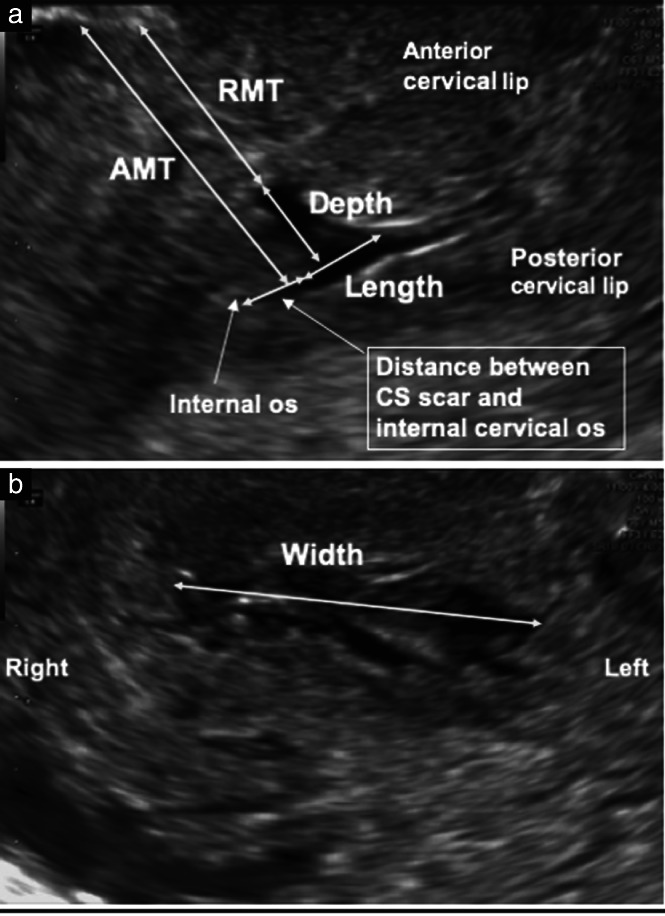
(a) Measurements of Cesarean section (CS) scar niche dimensions in the sagittal plane, including its largest length, largest depth, residual myometrial thickness (RMT) and adjacent myometrial thickness (AMT). (b) Largest width of the CS scar niche measured in the transverse plane.

The position of the CS scar was assessed in the sagittal plane and defined as the distance between the base of the CS scar and the level of the internal cervical os (Figure 1). The internal cervical os was identified by visualizing the whole cervical canal and using color Doppler mapping of the uterine artery in the paracervical region. When a niche was present, this distance was measured from the edge of the niche closest to the internal os (Figure 2a). Parameters of the niche evaluated in the sagittal plane included its largest length, largest depth, residual myometrial thickness (RMT) and adjacent myometrial thickness (AMT) (Figure 2a). The largest width of the niche was evaluated in the transverse plane (Figure 2b).

**Figure 3:**
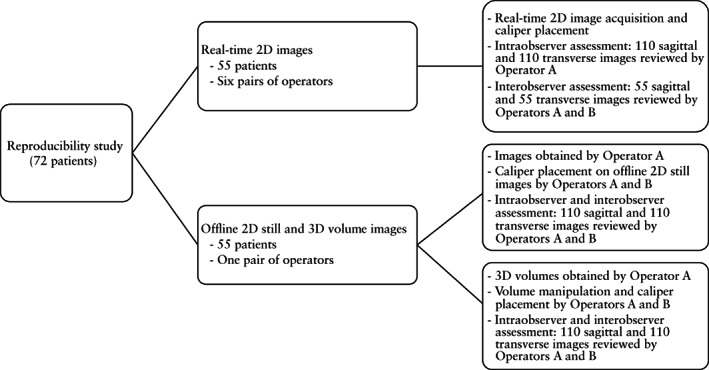
Flowchart summarizing the study design. 2D, two‐dimensional; 3D, three‐dimensional.

Reproducibility of assessment was studied using three sets of images (Figure 3). In the first set, two‐dimensional (2D) images were acquired, and calipers were placed independently in real time by two operators. In the second set, offline caliper placement was performed by two operators on stored 2D images acquired by one operator. In the third set, volumes were acquired by one operator, and manipulation of stored 3D volumes and caliper placement were performed by two operators on extracted 2D images.

To assess real‐time 2D image acquisition and caliper placement, two operators performed transvaginal ultrasound examination independently and were blinded to each other's findings, acquiring images and placing calipers independently in real time. To achieve this in a timely, blinded way, the first operator performed the scan with the second operator in the room but behind the curtain around the scanning couch; the second operator then took over scanning and repeated all the measurements while the first operator stepped behind the curtain. The second operator did not access images and caliper placement obtained by the first operator. The whole scan took a maximum of 3 min by each operator. To assess intraobserver reproducibility, the first operator acquired real‐time images twice.

To study caliper placement reproducibility on 2D offline still images, two operators placed calipers on offline stored images acquired by the first operator. On the stored images, only the internal cervical os was marked from real‐time image acquisition, and no calipers were placed or stored. Cervical length and CS scar measurements were recorded by each operator twice independently, on different days. Intraobserver reproducibility for caliper placement was assessed by comparing each operator's first and second measurements. To calculate the interobserver reproducibility, the first measurements of one operator were compared with those of the other operator; this was then repeated for the second measurements.

For the 3D images set, each operator manipulated the volume acquired by the first operator, extracted images and independently placed calipers for all measurements twice, on different days. Intraobserver and interobserver reproducibility were calculated using both measurements from both operators.

This new assessment protocol was approved as part of service evaluation and quality improvement by the hospital clinical governance, and introduced into routine clinical practice; therefore, ethical approval and individual patient written consent were not required. Verbal informed consent was obtained from patients prior to performing the transvaginal scan in accordance with current clinical guidelines.

Data were analyzed using SPSS version 26.0 (SPSS Inc., Chicago, IL, USA). Cohen's kappa was calculated to evaluate the agreement for categorical variables (i.e. CS scar visibility and presence of a niche). For Cohen's kappa, values between 0.81 and 1.0 were considered to show excellent agreement, while values of 0.61–0.80, 0.41–0.60, 0.21–0.40 and 0–0.20 indicated good, moderate, fair and poor agreement, respectively^17^.

A one‐sample Student's *t*‐test with a significance level of 0.05 was used to assess mean differences between measurements obtained by the same operator (intraobserver reproducibility) and different operators (interobserver reproducibility). Mean differences and 95% limits of agreement (LOA) were presented graphically and analyzed using Bland–Altman plots. Upper and lower 95% LOA are calculated in each case as mean difference ± 1.96 SD as reported in the figures. Outliers that were greater than 3 SD from the mean difference were excluded from the final analysis.

A power calculation was performed to evaluate the number of women required to identify optimal reproducibility values. We estimated that a total of 100 images would be required to detect significant differences between two operators, in accordance with calculations from previous studies^18^, ^19^, ^20^. As a previous study has shown that the CS scar could be visualized in 90% of cases^13^, 55 cases (110 sagittal and transverse images to achieve adequate power) were reviewed for each part of the study.

## RESULTS

Between May 2019 and December 2020, a total of 72 women with a singleton pregnancy and previous full‐dilatation CS were included in the study, and 550 images were reviewed for assessment of reproducibility. Two women had undergone a preterm full‐dilatation CS (at 31 and 33 weeks), whereas the remainder delivered by full‐dilatation CS at term (≥ 37 weeks' gestation). Three women in this cohort had had two previous CS, including a full‐dilatation CS. Only one CS scar was visible on ultrasound in these women and used for measurement of CS scar parameters. All other women had undergone one previous CS at full dilatation. Demographic characteristics of the included women are shown in Table S1.

**Figure 4:**
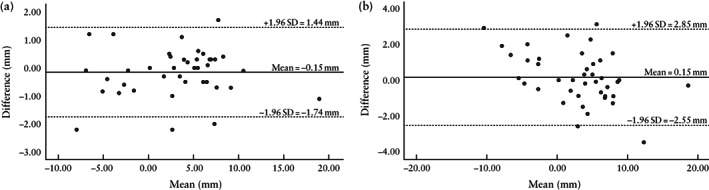
Bland–Altman plots showing intraobserver (a) and interobserver (b) reproducibility of measurements of distance from full‐dilatation Cesarean section scar to internal cervical os on real‐time two‐dimensional images.

For real‐time 2D images, kappa coefficients of 0.84 and 0.85 were obtained for CS scar visibility and the presence of a niche, respectively, indicating excellent interobserver agreement. Both operators were unable to see the CS scar in nine cases, and there was disagreement regarding visualization of the CS scar in two cases; the CS scar was therefore visualized in 80% (44/55) of cases by both operators. A niche was identified by both operators in 64% (28/44) of the visualized CS scars. There was good intraobserver reproducibility of measurement of cervical length, CS scar position and niche characteristics, with small mean differences (≤ 1.1 mm) and narrow 95% LOA (≤ ± 2.6 mm) (Table 1). The intraobserver difference for cervical length and distance from CS scar to the internal os was within 2 mm in > 95% of cases; for all other measurements of CS scar characteristics, the intraobserver difference was within 2 mm in > 80% of cases. Reproducibility of CS scar and niche measurements was the best for the distance from the CS scar to the internal cervical os, with intraobserver and interobserver 95% LOA of ± 1.59 and ± 2.70 mm, respectively (Figure 4). Interobserver reproducibility was lowest for measurement of the niche width, with 95% LOA of ± 5.78 mm (Table 2).

**Table 1:** Intraobserver reproducibility for Cesarean section (CS) scar and niche measurements using real‐time two‐dimensional (2D) images, offline 2D still images and three‐dimensional (3D) volume images

	Real‐time 2D images			Offline 2D still images			3D volume images		
Parameter	Mean difference (mm)	95% LOA (mm)*	Difference ≤ 2 mm (%)†	Mean difference (mm)	95% LOA (mm)*	Difference ≤ 2 mm (%)†	Mean difference (mm)	95% LOA (mm)*	Difference ≤ 2 mm (%)†
Cervical length	−0.09	± 1.49	98.1	−0.12	± 1.90	94.3	−0.05	± 1.43	99.1
Distance from CS scar to internal os	−0.15	± 1.59	95.5	0.05	± 1.14	100	0.18	± 1.82	94.6
CS scar niche									
Length	−0.37	± 2.00	92.9	0.00	± 1.69	95.6	0.08	± 2.45	84.7
Depth	−0.48	± 1.90	92.6	−0.03	± 1.63	97.1	−0.12	± 2.37	90.1
Width	−1.09	± 1.84	88.5	−0.22	± 1.86	93.8	0.10	± 3.59	76.1
RMT	−0.03	± 2.10	88.9	−0.12	± 1.61	98.5	−0.03	± 2.04	94.0
AMT	−0.09	± 2.57	81.5	−0.32	± 2.37	91.0	0.06	± 3.41	83.1

* Upper and lower boundaries of 95% limits of agreement (LOA) can be calculated in each case as mean difference ± value shown.

† Calculated as percentage of images included in final analysis.

AMT, adjacent myometrial thickness; RMT, residual myometrial thickness.

**Table 2:** Interobserver reproducibility for Cesarean section (CS) scar and niche measurements using real‐time two‐dimensional (2D) images, offline 2D still images and three‐dimensional (3D) volume images

	Real‐time 2D images			Offline 2D still images			3D volume images		
Parameter	Mean difference (mm)	95% LOA (mm)*	Difference ≤ 2 mm (%)†	Mean difference (mm)	95% LOA (mm)*	Difference ≤ 2 mm (%)†	Mean difference (mm)	95% LOA (mm)*	Difference ≤ 2 mm (%)†
Cervical length	0.13	± 3.51	76.4	−0.28	± 3.55	78.5	0.22	± 3.80	74.1
Distance from CS scar to internal os	0.15	± 2.70	86.4	0.38	± 2.00	94.4	−0.38	± 3.92	82.6
CS scar niche									
Length	−0.12	± 3.59	75.0	0.52	± 3.84	79.1	−0.22	± 3.65	75.8
Depth	−0.70	± 3.96	71.4	0.60	± 3.37	76.5	1.22	± 4.00	66.7
Width	0.36	± 5.78	56.0	1.32	± 3.90	64.6	1.98	± 6.25	32.8
RMT	0.07	± 3.25	76.9	0.43	± 3.08	83.1	0.22	± 3.80	74.6
AMT	−0.49	± 3.59	72.0	0.71	± 4.92	63.6	−0.10	± 4.92	66.7

* Upper and lower boundaries of 95% limits of agreement (LOA) can be calculated in each case as mean difference ± value shown.

† Calculated as percentage of images included in final analysis.

AMT, adjacent myometrial thickness; RMT, residual myometrial thickness.

For offline 2D still images, kappa coefficients of 0.71 and 0.73 were obtained for CS scar visibility and the presence of a niche, respectively, indicating good interobserver agreement. As expected, agreement was lower when using offline 2D still images compared with real‐time 2D images, demonstrating the added benefit of each operator assessing the CS scar and acquiring images themselves; however, reassessment of the CS scar on still images for quality control is feasible^21^. The CS scar was visualized by both operators in 84% (46/55) of cases, and a niche was identified in 74% (34/46) of cases. Intraobserver and interobserver reproducibility was again noted to be highest for measurement of the distance from the CS scar to the internal cervical os, with 95% LOA of ± 1.14 and ± 2.00 mm, respectively. Quantitative reproducibility values for offline 2D still images were narrower compared with those for real‐time images. In terms of intraobserver reproducibility, distance from the CS scar to the internal os, niche length, niche depth and RMT had a difference within 2 mm in > 95% of cases, while cervical length, niche width and AMT were within 2 mm in > 90% of cases. When comparing interobserver reproducibility of distance from the CS scar to the internal cervical os for the two 2D groups, caliper placement accounted for 74% of the reproducibility.

For 3D volume images, kappa coefficients of 0.69 and 0.67 were obtained for CS scar visibility and presence of a niche, respectively, indicating good interobserver agreement; however, these reproducibility values were lower compared with those for 2D images. Both operators visualized the CS scar in 84% (46/55) of cases, and a niche was identified in 72% (33/46) of cases. Both operators were able to determine the appropriate plane for measurement of the niche width in all cases with an identifiable niche; this result was in contrast to that for real‐time 2D images, in which 10.7% of niche width measurements were not obtained by either operator due to the difficulty of assessing the appropriate plane. The 95% LOA for intraobserver and interobserver reproducibility were generally slightly wider for 3D volume images than for 2D images (Tables 1 and 2). Overall, intraobserver and interobserver 95% LOA were narrower for cervical length, CS scar distance to the internal os, niche length, niche depth and RMT (≤ ± 2.5 and ≤ ± 4.0 mm, respectively) compared with niche width and AMT (≤ ± 3.6 and ≤ ± 6.3 mm, respectively). As noted with real‐time 2D reproducibility, interobserver 95% LOA were the widest for niche width measurement (± 6.25 mm). Figure S1 shows the Bland–Altman plots for intraobserver and interobserver reproducibility for each evaluated parameter and each set of images.

## DISCUSSION

In this study, we have tested the reproducibility of a midtrimester transvaginal ultrasound protocol for assessing the position of the CS scar and scar niche in pregnant women with a previous full‐dilatation CS. The CS scar could be visualized by both operators in ≥ 80% of cases in all three sets of images: real‐time 2D, offline 2D still and 3D volume images. There was a high level of agreement between operators for CS scar visibility and the presence of a niche. CS scar measurements had a high level of intraobserver and interobserver reproducibility for both 2D and 3D imaging. It can be difficult to differentiate between the internal cervical structure and the lower uterine segment. We therefore used color Doppler and uterine artery visualization in the paracervical region to determine the level of the internal os and found that measurement of the distance from the CS scar to the internal cervical os using this method was highly reproducible. Caliper placement on 2D still images had the narrowest intraobserver and interobserver 95% LOA for most parameters. Measurement of the niche width in the transverse plane had the widest interobserver 95% LOA for real‐time 2D and 3D volume images. Although 3D volume reproducibility was mostly comparable to that of 2D measurements, overall, assessment was more reproducible for 2D than 3D images; however, the latter may be used for offline quality control purposes^22^, ^23^.

To the best of our knowledge, only one reproducibility study has been performed on ultrasound assessment of CS scar niche characteristics during the second trimester of pregnancy^13^. In contrast to our study, all measurements in that study were carried out offline, retrospectively, using only stored images. In addition, the study did not assess the reproducibility of assessment of CS scar position relative to the internal os and included any woman with a singleton pregnancy who had at least one previous lower‐segment CS. For second‐trimester assessment of niche length, depth, width and RMT, the interobserver 95% LOA were all ≤ 4.0 mm, which is comparable to our interobserver reproducibility for offline 2D still images. Similar to our study, both Naji *et al*. and, more recently, Savukyne *et al*. reported CS scar detection rates of 88.8% and 77.9%, respectively, during pregnancy, with excellent agreement for CS scar and niche visibility^13^, ^14^. Zimmer *et al*. demonstrated that the ability to visualize the CS scar was significantly greater with increasing cervical dilatation at the time of CS^24^. As our cohort of patients included only women with a previous full‐dilatation CS, this may also explain the high level of intraobserver and interobserver reproducibility.

Two recent studies on reproducibility of 3D ultrasound measurement of a CS scar niche have been performed in non‐pregnant women, but they did not report on CS scar location relative to the internal os^25^, ^26^. Glavind *et al*. found intraobserver and interobserver 95% LOA of ≤ 4.0 mm for niche parameters^26^. This was similar to our 3D volume reproducibility values, although we noted wider interobserver 95% LOA for niche width and AMT measurements. Bij de Vaate *et al*. found good reproducibility of measurements of the niche in the longitudinal plane; however, similar to our study, the 95% LOA were wide in the transverse plane for width measurement (± 6.4 mm for niche width at base)^25^.

A strength of our study is that we evaluated interobserver reproducibility of assessment of real‐time 2D images within our routine clinical setting. The operators were experienced in assessing CS scars. We developed a protocol for how to perform all measurements in a standardized way. This approach has already been demonstrated to be reproducible in fetal biometry and may represent a tool for future research^27^, ^28^. For real‐time 2D reproducibility, six operator pairs were involved compared with a single pair of operators for offline 2D still and 3D volume reproducibility assessments. This may have had an impact on the results. However, we consider that real‐time reproducibility assessed among multiple operators reflects clinical practice more accurately, making the findings robust for clinical translation. 2D measurements were more reproducible than 3D measurements. Similar findings have been reported when comparing reproducibility of 2D and 3D measurements for fetal biometry^29^. It is unlikely that this was related to training, as all operators were experienced in scanning and underwent a standardization session for both 2D and 3D imaging^30^.

Only two women included in the study had a previous preterm full‐dilatation CS, and three women had two previous CS, for which only one scar was visualized. It is not possible to ascertain whether the scar was from the full‐dilatation CS. Vikhareva Osser *et al*. have reported that multiple scars can be difficult to visualize separately^31^. We consider it unlikely that these limitations had a significant impact on the visibility of CS scars in our study.

A screening test needs to be both feasible and reproducible. Our findings indicate that assessment of CS scar characteristics is highly reproducible in the second trimester of pregnancy. The reproducibility of assessment may have important clinical implications for examining women with a previous full‐dilatation CS and predicting their risk of subsequent preterm birth. The most reproducible CS scar measurements were the distance from the CS scar to the internal cervical os, and niche length, depth and RMT.

The optimal management of subsequent pregnancies in women with a history of full‐dilatation CS is currently unknown. Among women with a prior late miscarriage/spontaneous preterm birth, placement of a vaginal cervical cerclage appears to be less effective in preventing preterm birth in those with a history of term full‐dilatation CS compared to those whose first term delivery was vaginal^32^. Therefore, assessing the location and parameters of the CS scar could enhance therapeutic options. This is currently the subject of an ongoing research trial^33^.

In conclusion, this study evaluated the intraobserver and interobserver reproducibility of assessment of CS scar visibility, position and niche measurements during the second trimester of pregnancy in pregnant women with a previous full‐dilatation CS. Our study shows a high level of reproducibility when assessing both 2D and 3D ultrasound images. The most reproducible CS scar measurement was the distance from the CS scar to the internal cervical os. CS scar measurements need to be assessed further in prospective studies to develop multivariable screening models for the prediction of spontaneous preterm birth.

## Supporting information


**Videoclip S1** Ultrasound protocol for measuring cervical length and Cesarean section scar characteristics in pregnancy.

**Appendix S1** Ultrasound protocol for assessment of cervical length and Cesarean section scar characteristics in pregnancy

**Table S1** Demographic characteristics of women with previous full‐dilatation Cesarean section who were included in the study

**Figure S1** Bland–Altman plots of intraobserver and interobserver reproducibility for each evaluated parameter and each set of images. AMT, adjacent myometrial thickness; CS, Cesarean section; RMT, residual myometrial thickness.

## Data Availability

The data that support the findings of this study are available from the corresponding author upon reasonable request.
