# Supplementary material for: Reproducibility of assessment of full‐dilatation Cesarean section scar in women undergoing second‐trimester screening for preterm birth
Source: Ultrasound Obstet Gynecol. 2022 Sep 1;60(3):396–403. doi: 10.1002/uog.26027 (PMC9545619; doi:10.1002/uog.26027)
Supplement: Supplementary file 2 — Appendix S1 Ultrasound protocol for assessment of cervical length and Cesarean section scar characteristics in pregnancy [file UOG-60-396-s004.docx]

**APPENDIX: Ultrasound protocol for assessment of cervical length and caesarean section scar characteristics in pregnancy**

**Ultrasound Definitions**

**Caesarean section scar -** hypoechogenic (or rarely hyperechogenic) discontinuity in the myometrium at the anterior wall of the lower uterine segment or cervix.

**Caesarean section niche -** an indentation at the site of the caesarean section (CS) scar with a depth of at least 2mm.

**Machine**

- Voluson E8 Expert ultrasound system (GE Healthcare, Zipf, Austria)
- 4–9-MHz transvaginal vaginal probe

**General ultrasound principles**

1. Obtain informed consent and offer chaperone
2. Women should have an empty bladder
3. Check for allergies to latex
4. Clean the transvaginal ultrasound probe
5. Cover the probe with ultrasound gel
6. Place a disposable sterile cover on the probe and apply sterile lubricating gel
7. Place women in the dorsal lithotomy position
8. Settings – routine obstetric setting with application set to cervix
9. Depth should be set where a panoramic view of the lower uterine segment can be obtained, including the cervical canal up to the external os.
10. The angle width and the magnification should be set to full range, where the axis of the cervical canal can be demonstrated in relation to the lower uterine segment.

**Protocol for measurements**

- **Measurement of cervical length**
- Gently advance the transvaginal probe slowly into the vagina looking at the image as the probe advances.
- Obtain a sagittal section of the uterus and cervix with good visualisation of the cervical canal.
- Excessive pressure on the cervix by the probe should be avoided. The anterior and posterior lips of the cervix should be of similar diameter.
- Use zoom to enlarge the view of the cervix. The cervix should occupy approximately 75% of the image.
- Identify the cervical canal, endocervical mucosa, internal os and external os,
- Use the uterine arteries and the endocervical mucosa as a guide to the relative position of the internal cervical os.
  - To identify the uterine artery:
    - Apply color flow mapping
    - Move the transducer laterally to view the para-cervical region
    - Gently move the transducer from one side to the other side, to identify the uterine artery and the level at which it straightens out (aliasing vessels coursing along the side of the cervix and uterus)
    - The internal os is at this level of the uterine arteries
- Using calipers measure the linear distance between the external and internal os. (Figure 1)
- Check for presence of funnelling or sludge
- At least 3 measurements should be obtained over a period of about 3 - 5 minutes in order to recognise any dynamic changes and the best shortest measurement of the cervical length recorded.
- Gently press on the fundus or the suprapubic area to assess for pressure effect

**Figure 1:** Measurement of cervical length

- **Measurement of CS scar position and niche characteristics**
  - To measure the CS scar position and niche, there should be good visualisation of the lower uterine segment and cervix. Scan across in the sagittal plane to identify the CS scar. After identifying the CS scar, magnify the image so that the scar occupies at least 50% of the image to ensure consistent and accurate measurements.
  - In the sagittal plane measure the distance between the base of the CS scar and the internal cervical os (in presence of a niche, measure the distance from the edge of the niche closest to the internal os). Record measurements above the internal cervical os as positive measurements and below it as negative measurements. CS scars at the internal os are noted as being at 0mm. (Figure 2)
  - Measurements of niche in sagittal plane (Figure 3): largest length, largest depth, residual myometrial thickness (RMT) and adjacent myometrial thickness (AMT).^1,2^

RMT - thinnest point of RMT should be measured (measured perpendicular to serosa)

AMT - measured next to the niche, where myometrium is thickest.

- To obtain the optimal sagittal plane - start with good visualisation of the cervical canal in the midsagittal plane and then slowly scan across laterally to both sides to identify the largest area of the niche.
  - Measurement of niche in the transverse plane (Figure 3): largest width
  - To obtain the optimal transverse plane – keep good visualisation of niche in the sagittal plane and slowly rotate the probe to transverse plane.
  - The CS scar and presence of niche is classified overall as visible, not visible or unclear.

**Figure 2:** Measurement of distance of caesarean scar to internal cervical os: CS scar above cervix (left). CS scar in cervix (right).

**Figure 3:** Measurements of niche in sagittal plane (left): largest length, largest depth, residual myometrial thickness (RMT) and adjacent myometrial thickness (AMT). Measurement of niche in the transverse plane (right): largest width.

- **Three-dimensional volume measurements**
- To record the volume, obtain a clear sagittal view of the lower uterine segment and the cervix where the internal os, the cervical canal and the external os are all seen at the same time within the three-dimensional sector. Set quality to maximum and sweep angle to 120 degrees. Obtain the volume by holding the transducer stationary and store images for offline analysis.
- Volume manipulation
  - Open volume in the multiplanar mode, where in accordance with the acquisition, the sagittal view is displayed in Plane A, and the transverse and coronal views in Planes B and C, respectively.
  - By rotating around the x- and y-axis obtain the optimal midsagittal section of the cervix and lower uterine segment. Examine the lower uterine segment and cervix to identify the CS scar and niche. The section with the largest area of the niche is used for measurements. Magnify the image as necessary and the measurements in the sagittal plane are performed as described above in the two- dimensional (2D) plane. With the niche visible in the sagittal plane, place the marker at the base of the niche and obtain the transverse plane (B plane) to assess and measure the largest width (Figure 4).


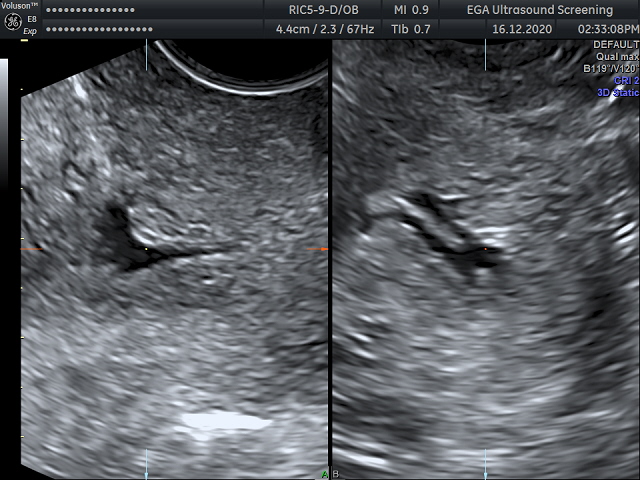


**Figure 4:** Volume imaging: Plane A – sagittal (left) and Plane B - transverse (right) showing CS scar and niche.

REFERENCES

1. Naji O, Abdallah Y, Bij De Vaate AJ, Smith A, Pexsters A, Stalder C, McIndoe A, Ghaem-Maghami S, Lees C, Brölmann HAM, Huirne JAF, Timmerman D, Bourne T. Standardized approach for imaging and measuring Cesarean section scars using ultrasonography. Ultrasound Obstet Gynecol. 2012;39(3):252–9.

2. Jordans IPM, de Leeuw RA, Stegwee SI, Amso NN, Barri-Soldevila PN, van den Bosch T, Bourne T, Brölmann HAM, Donnez O, Dueholm M, Hehenkamp WJK, Jastrow N, Jurkovic D, Mashiach R, Naji O, Streuli I, Timmerman D, van der Voet LF, Huirne JAF. Sonographic examination of uterine niche in non-pregnant women: a modified Delphi procedure. Ultrasound Obstet Gynecol. 2019;53(1):107–15.
