# Supplementary material for: Reproducibility of assessment of full‐dilatation Cesarean section scar in women undergoing second‐trimester screening for preterm birth
Source: Ultrasound Obstet Gynecol. 2022 Sep 1;60(3):396–403. doi: 10.1002/uog.26027 (PMC9545619; doi:10.1002/uog.26027)
Supplement: Supplementary file 3 — Table S1 Demographic characteristics of women with previous full‐dilatation Cesarean section who were included in the study [file UOG-60-396-s001.docx]

**Table S1** Demographic characteristics of women with previous full dilatation CS who were included in the study

| **Characteristics** |  |
| --- | --- |
| Maternal age, years | 35.1 (±4.7) |
| BMI, kg/m^2^ | 25.3 (±4.5) |
| GA at scan (weeks) | 17.9 (± 2.6) |
| Ethnicity |  |
| White | 48 (66.7%) |
| Black | 6 (8.3%) |
| South East Asian | 11 (15.3%) |
| Others | 7 (9.7%) |

Data are given as mean (± SD) or n (%). BMI, body mass index; GA, gestational age.
