# Supplementary material for: Reproducibility of assessment of full‐dilatation Cesarean section scar in women undergoing second‐trimester screening for preterm birth
Source: Ultrasound Obstet Gynecol. 2022 Sep 1;60(3):396–403. doi: 10.1002/uog.26027 (PMC9545619; doi:10.1002/uog.26027)

**Supplementary figure 1:** Bland-Altman plots of intra- and interobserver reproducibility.

CS (caesarean section); RMT (residual myometrial thickness); AMT (adjacent myometrial thickness).

**(a) Reproducibility for image acquisition and caliper placement on two-dimensional (2D) images, acquired by two operators in real time at the same time: ‘Real-time 2D images’**


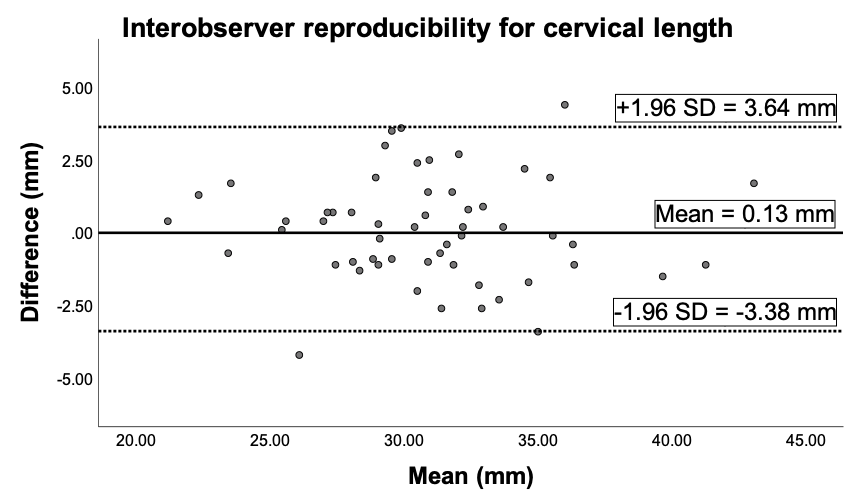

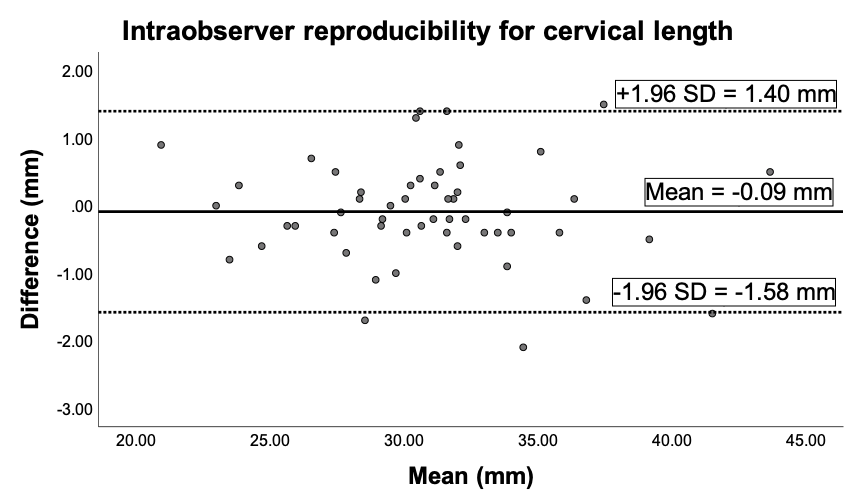


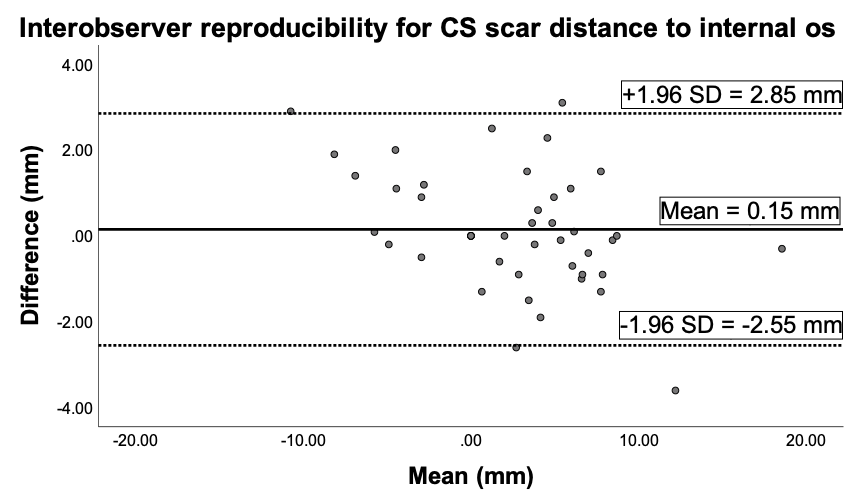

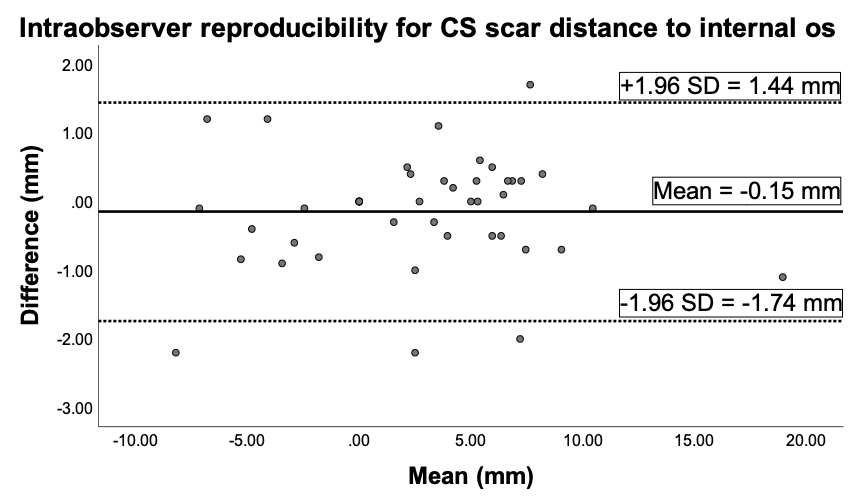


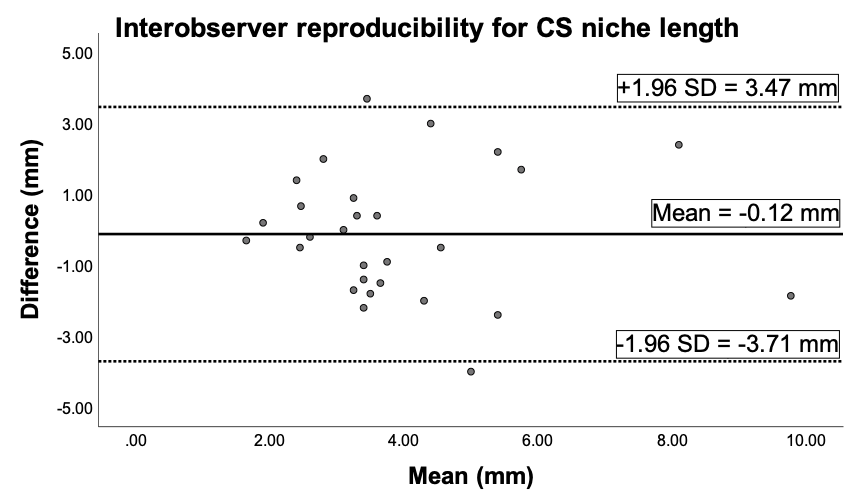

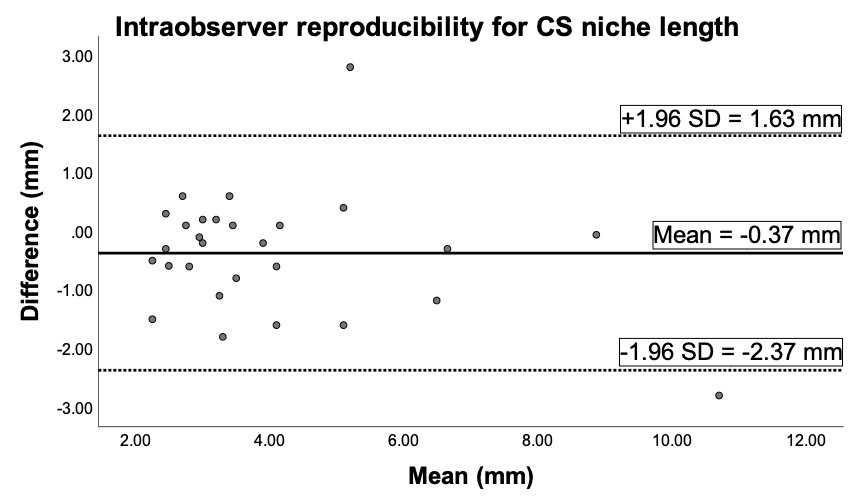


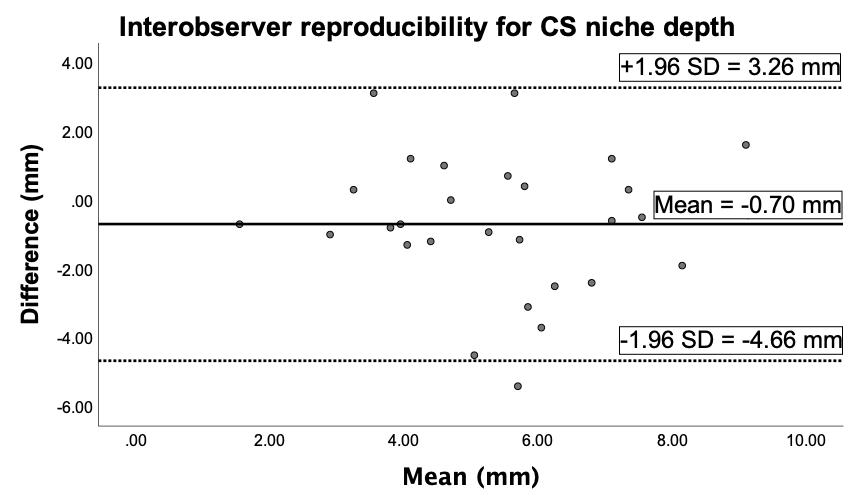

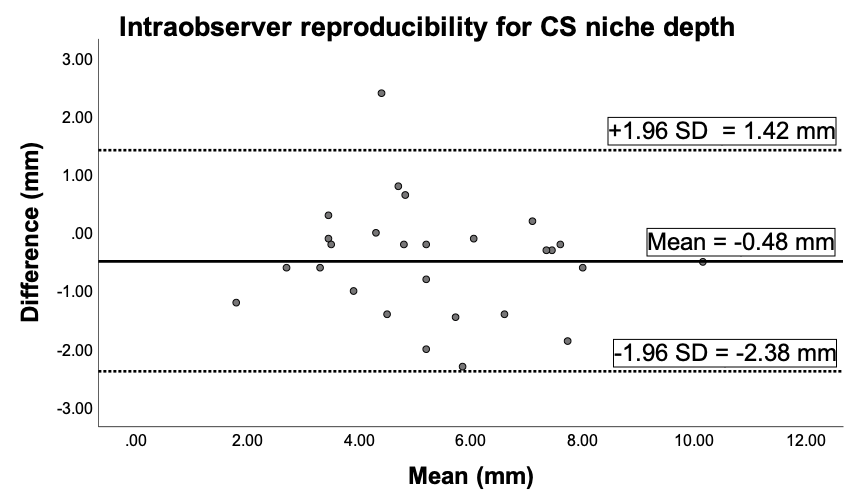


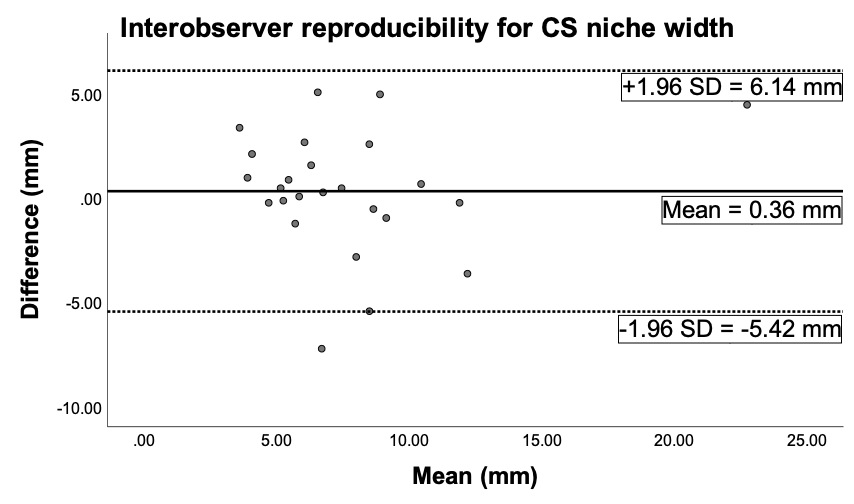

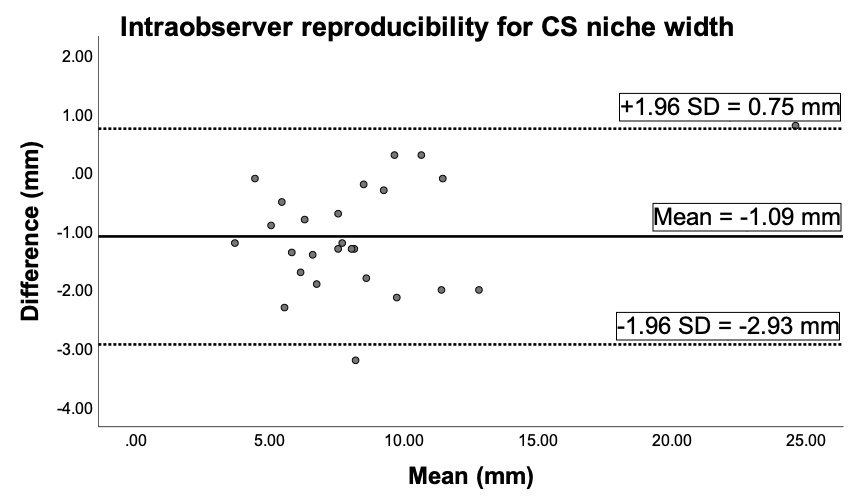


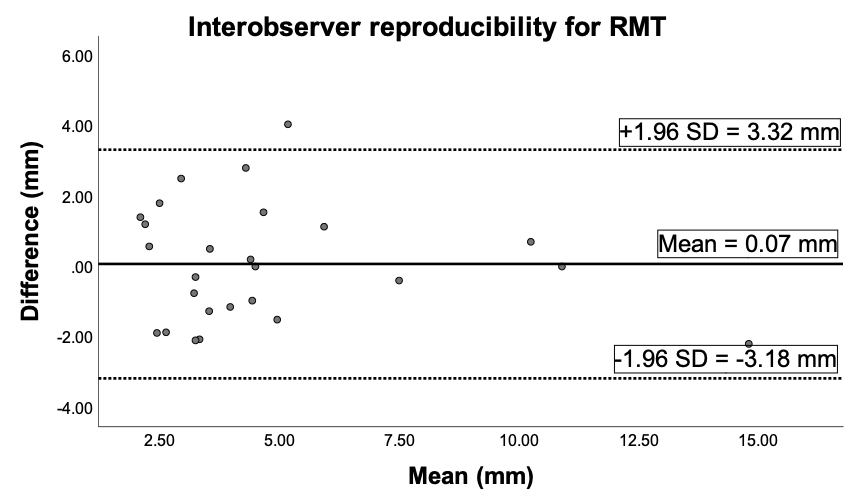

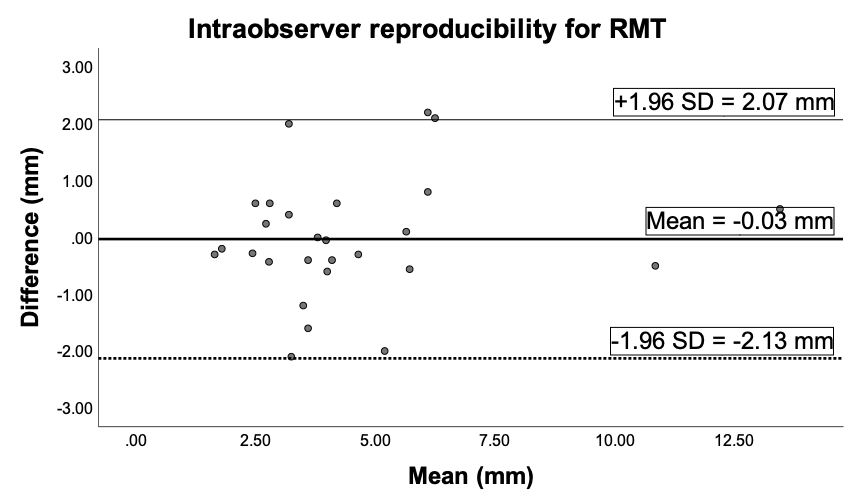


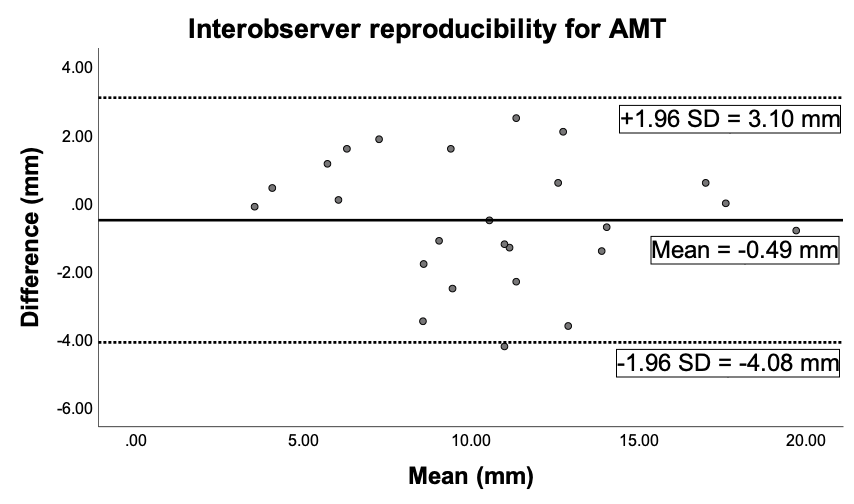

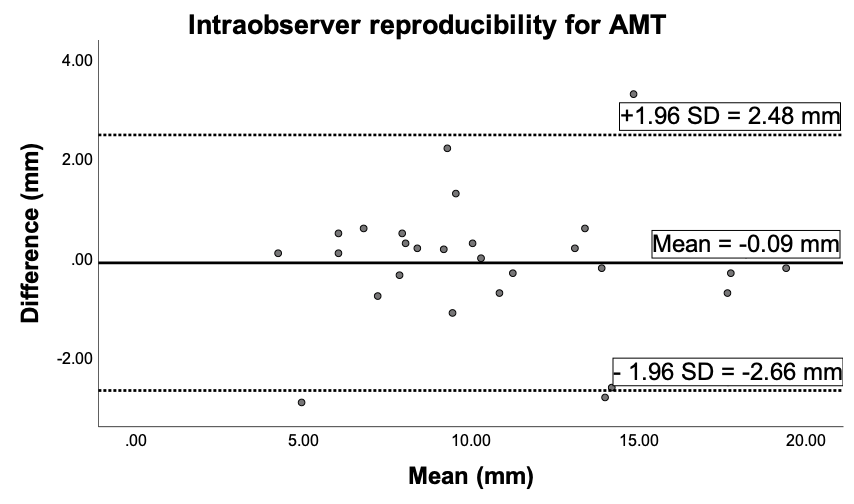


**(b):** **Reproducibility for offline caliper placement by two operators on stored 2D images acquired by the first operator: ‘Offline 2D still images’**


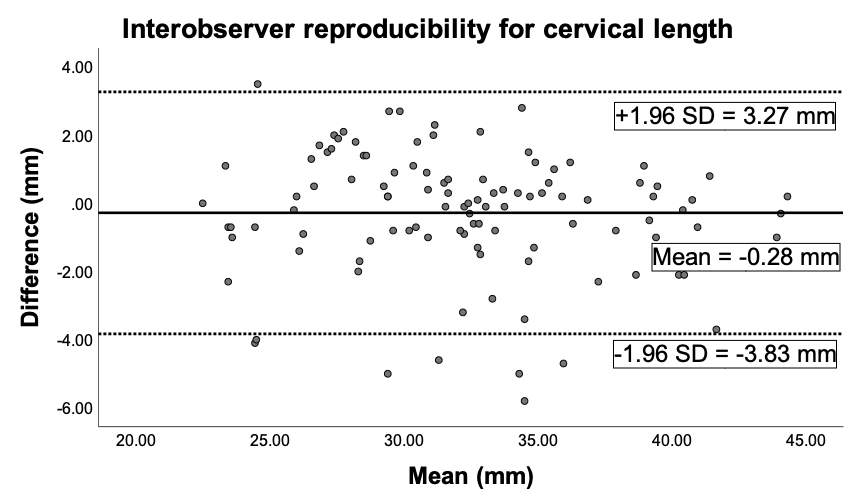

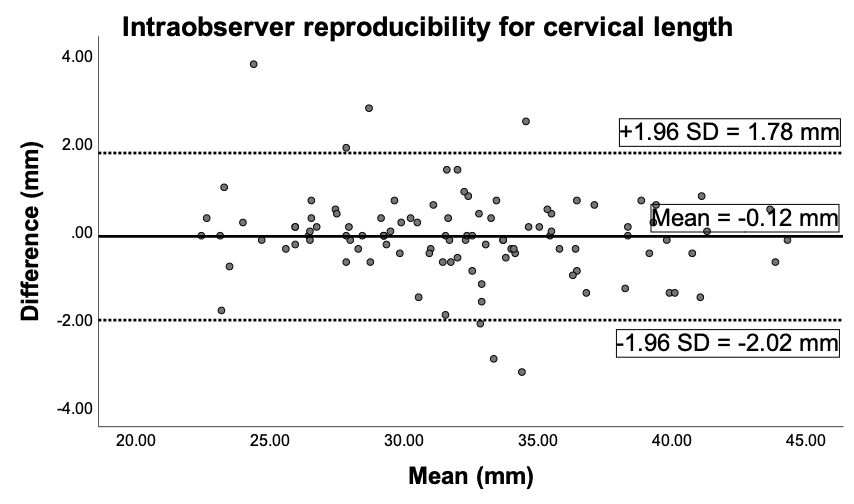


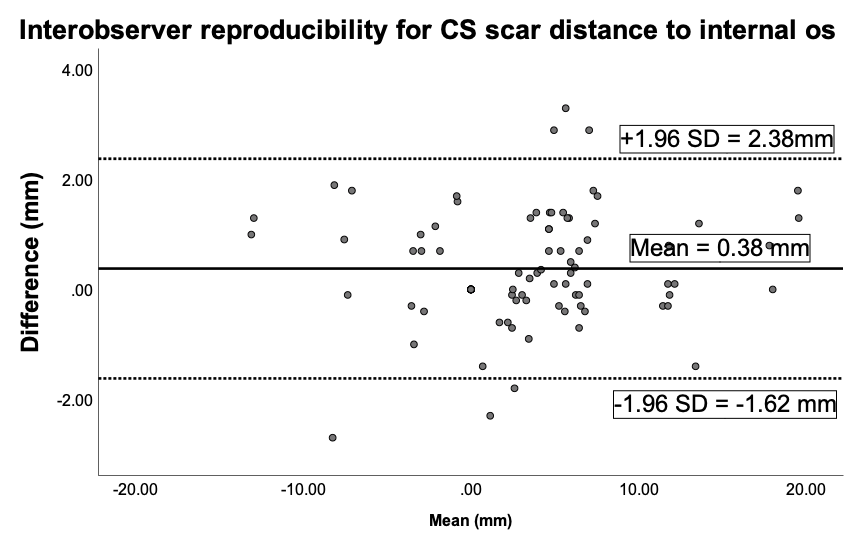

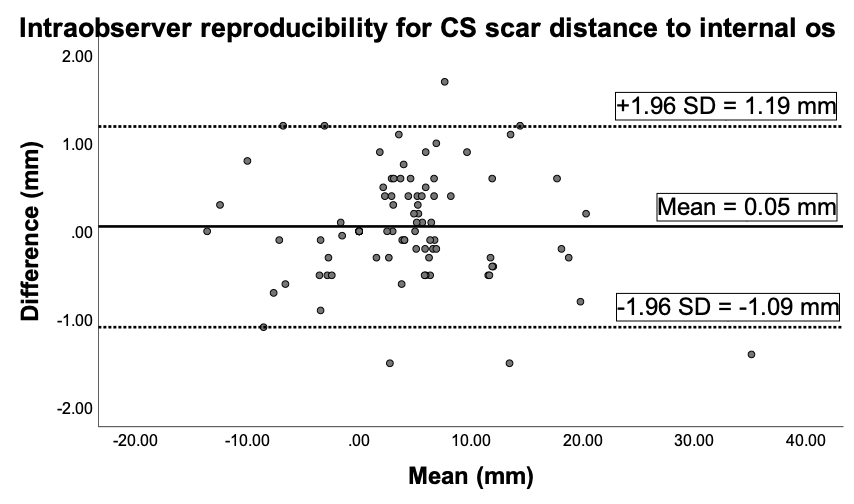


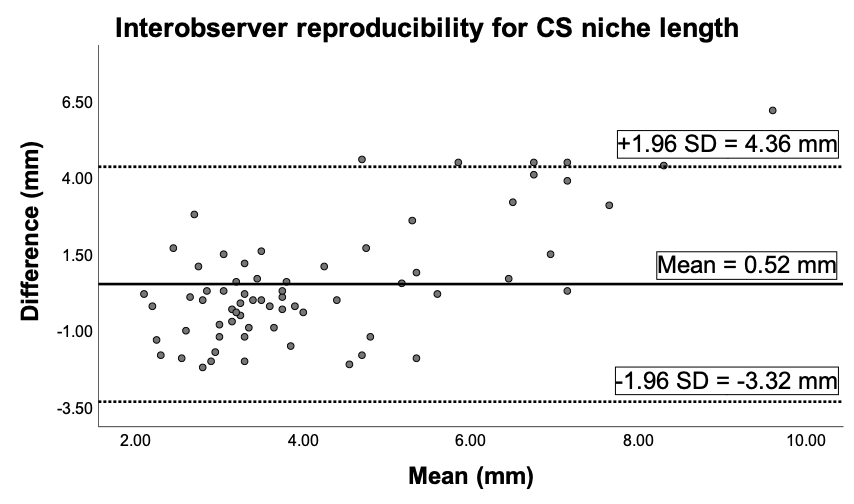

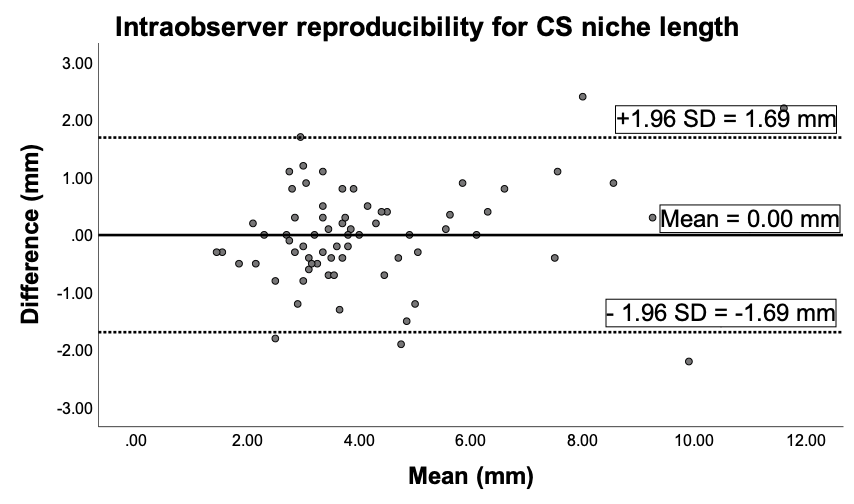


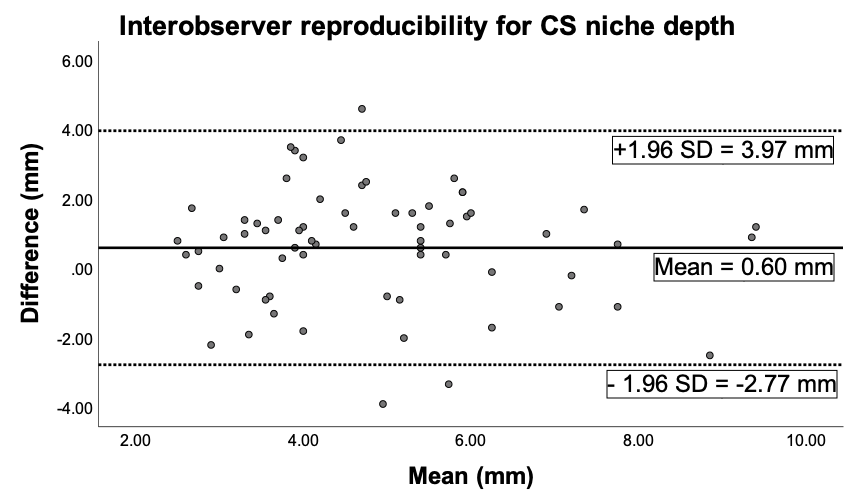

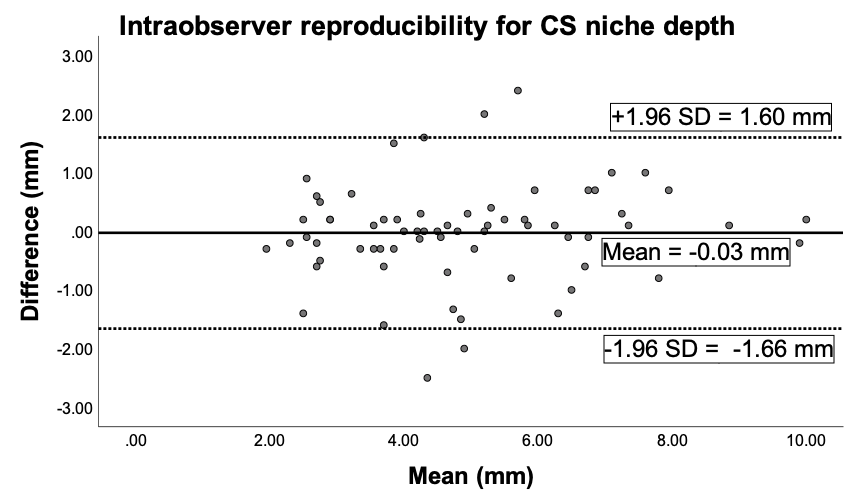


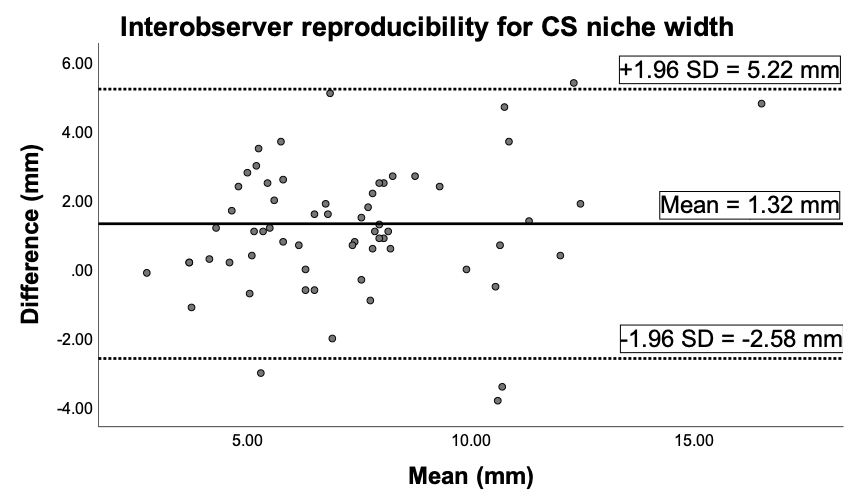

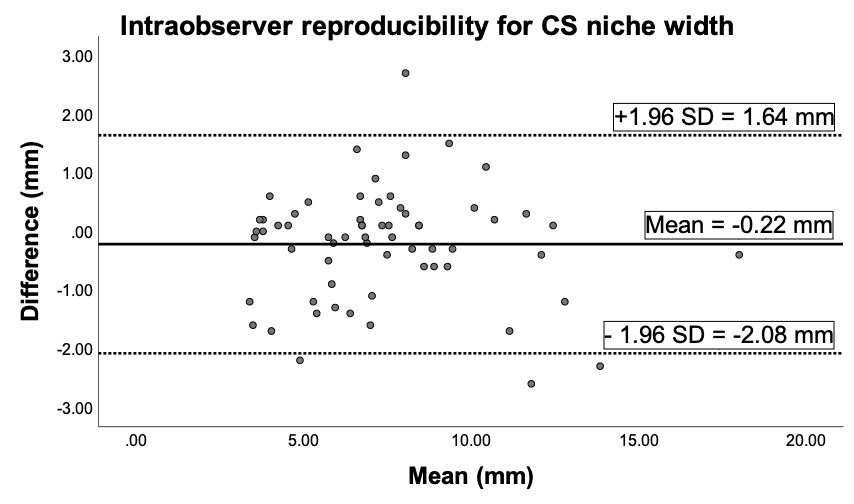


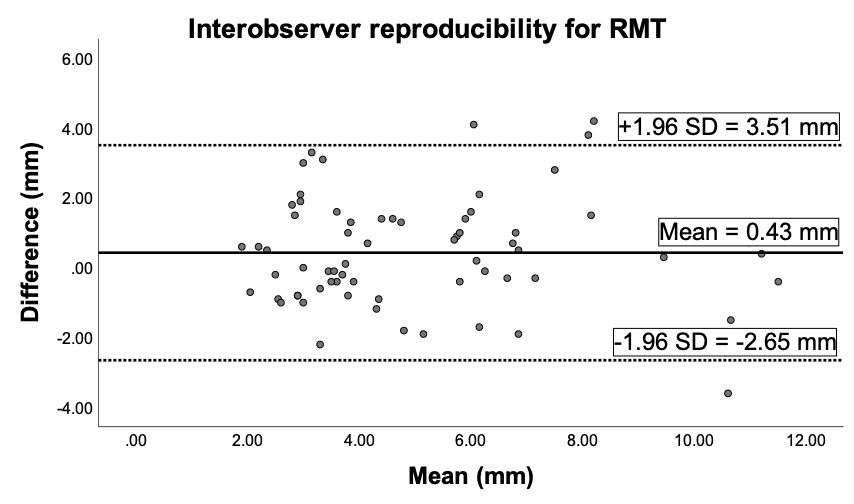

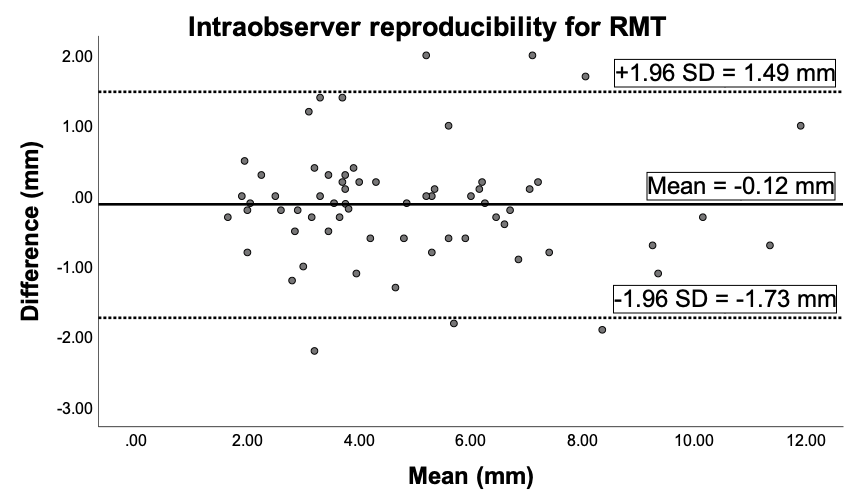


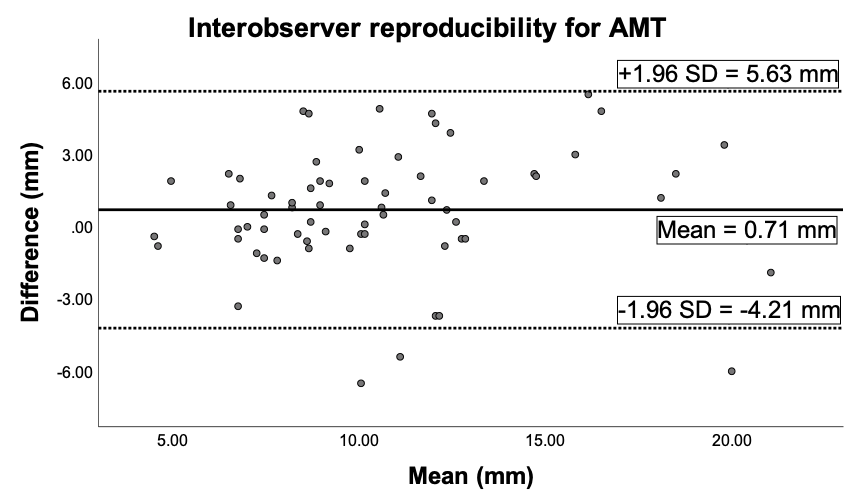

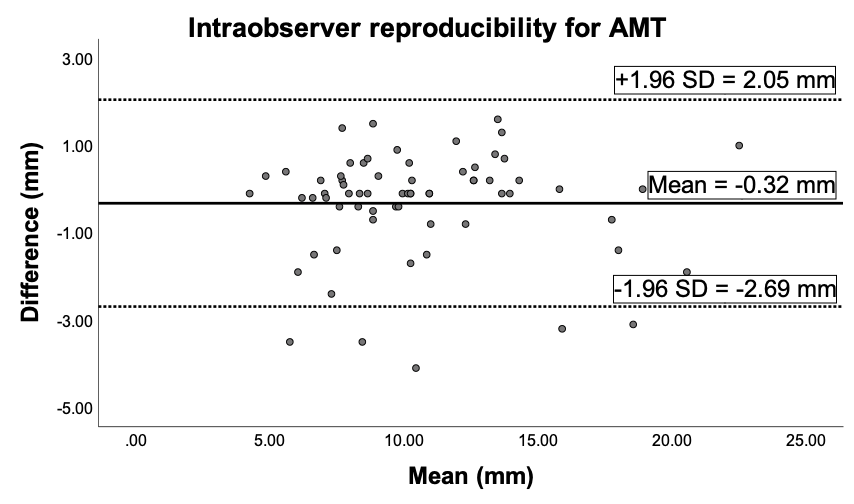


**(c):** **Reproducibility for stored three-dimensional volume manipulation and caliper placement on extracted 2D images by two operators. Volumes were acquired by the first operator: ‘3D volume images’.**


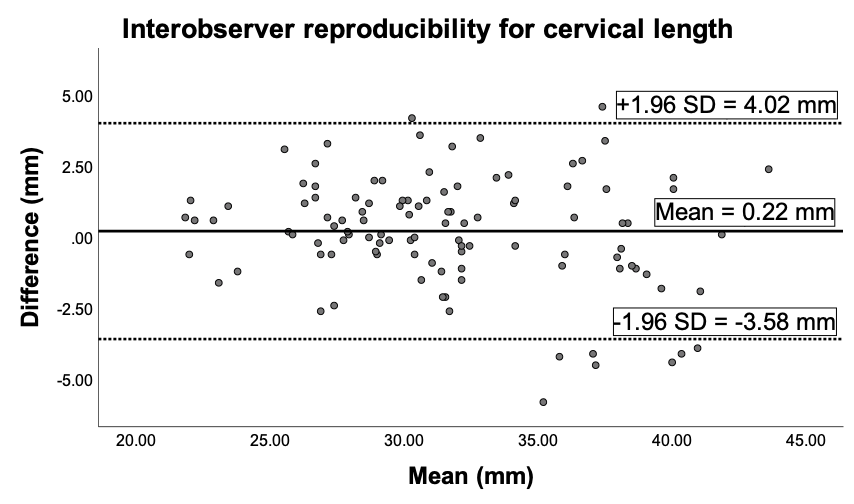

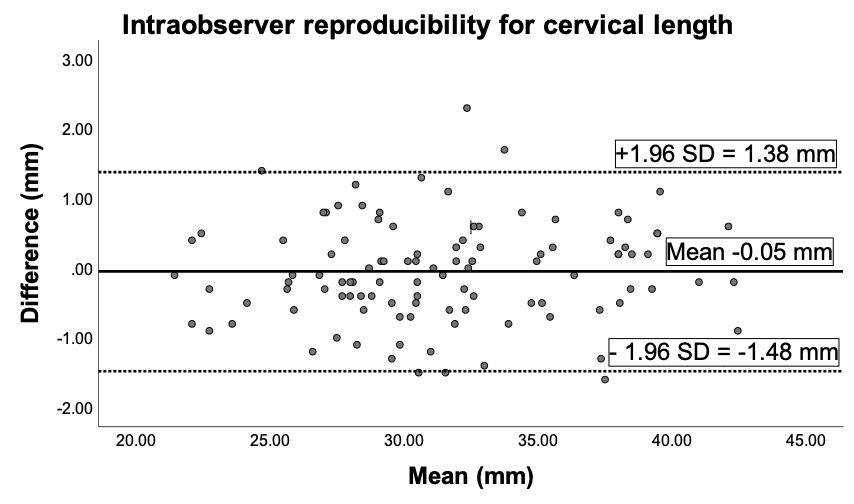


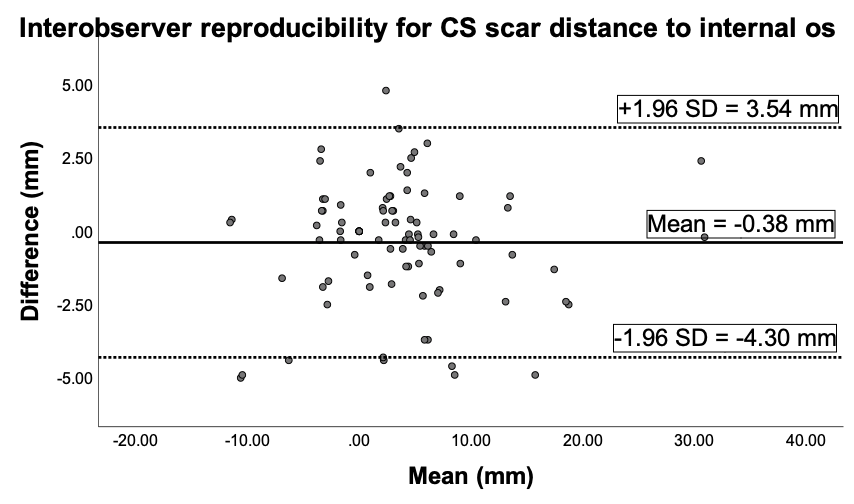

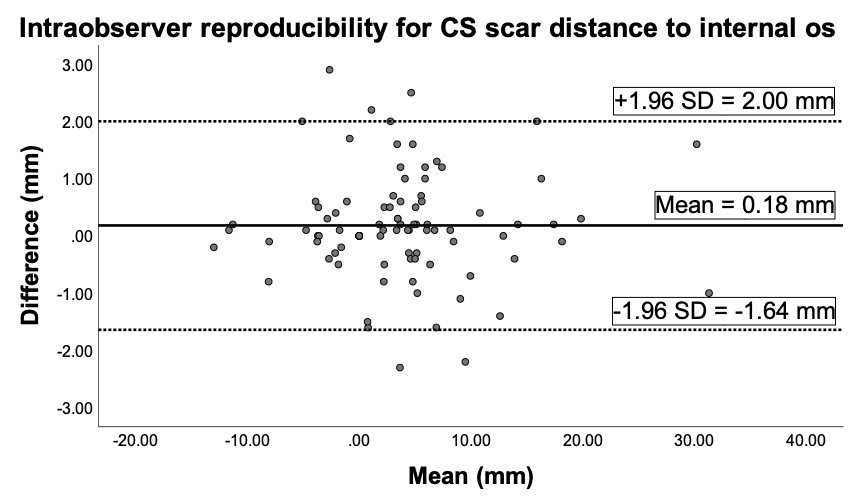


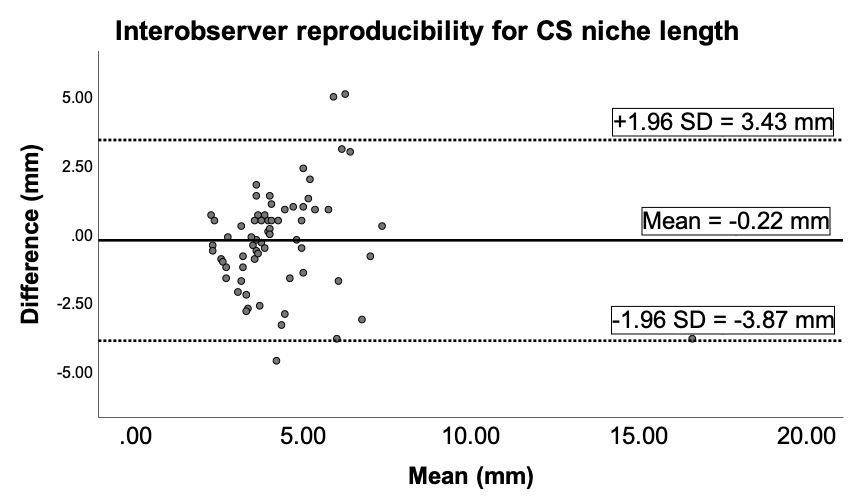

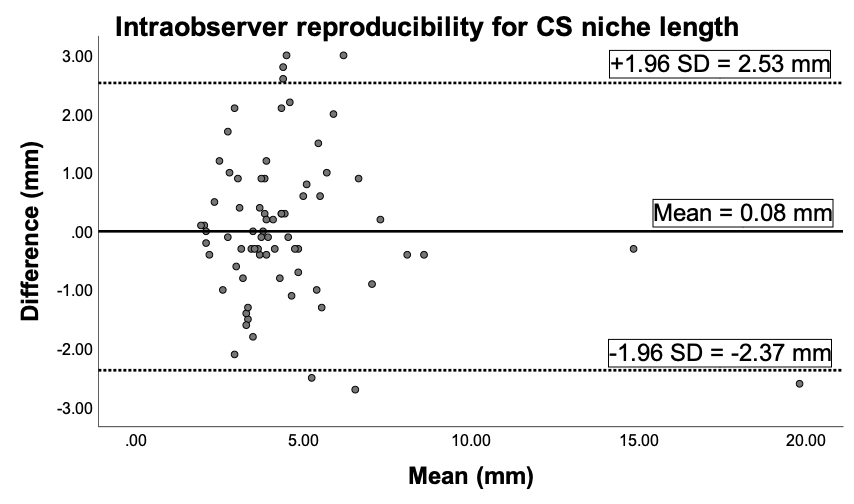


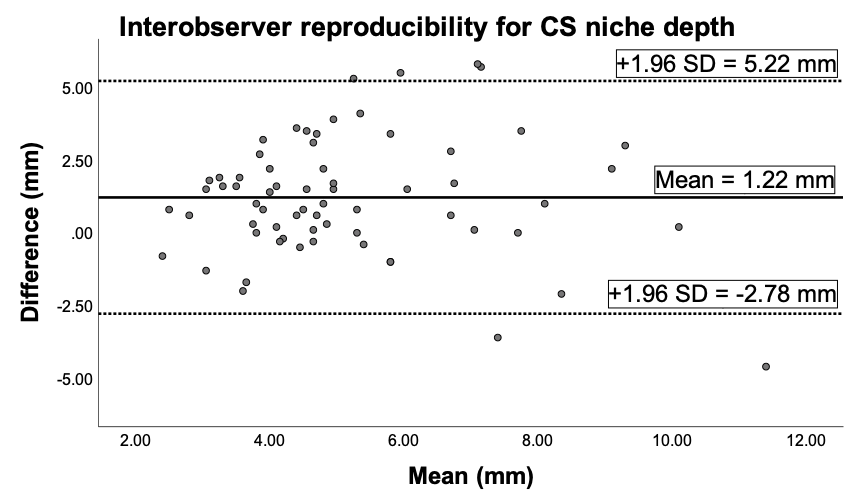

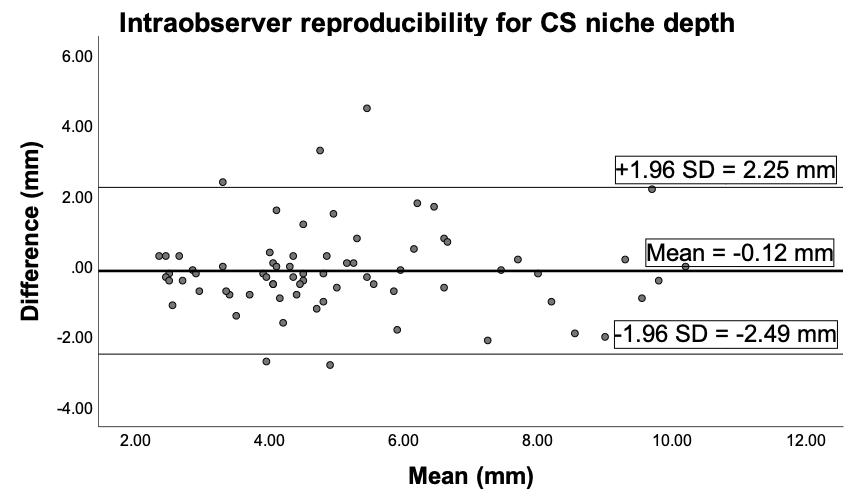


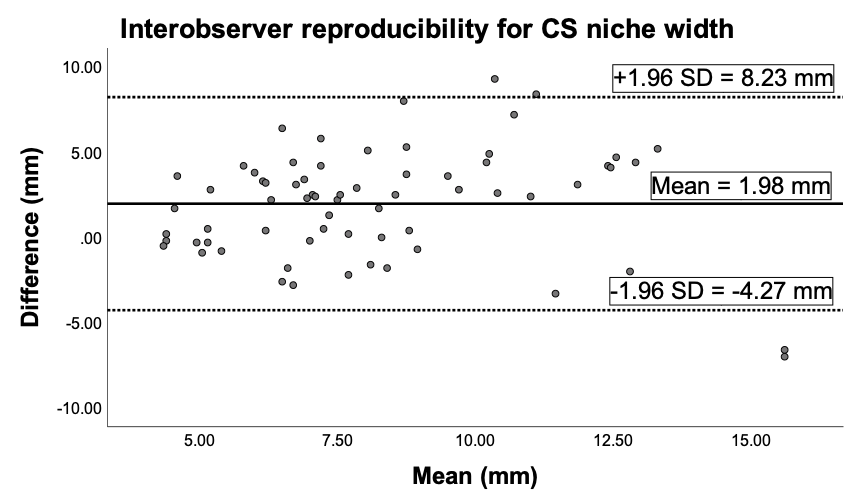

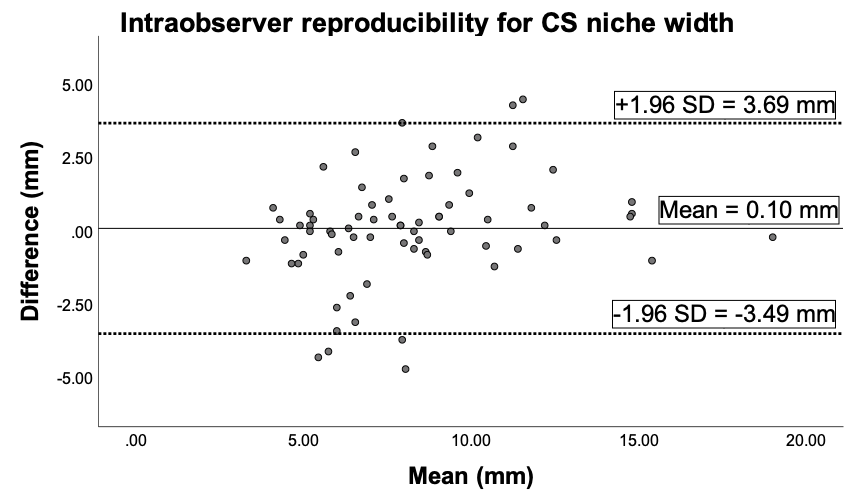


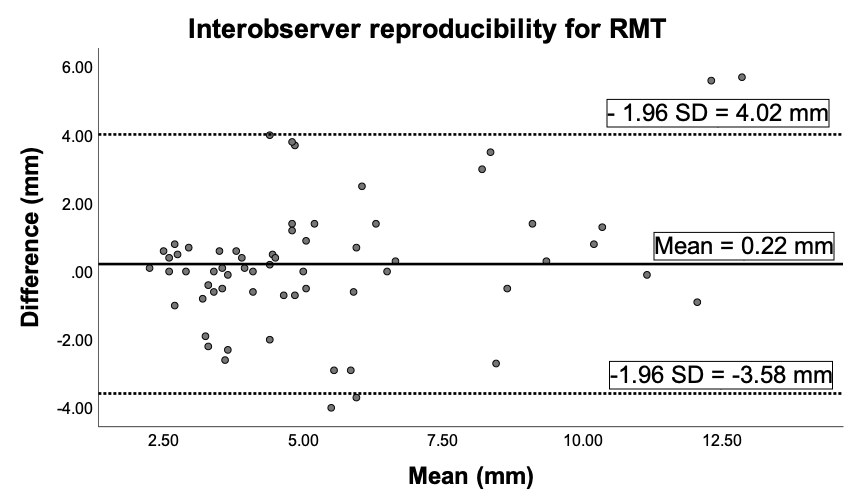

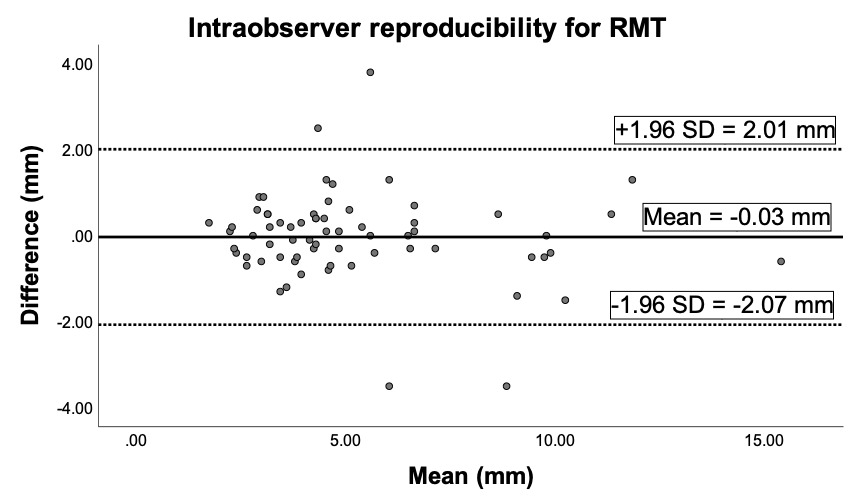


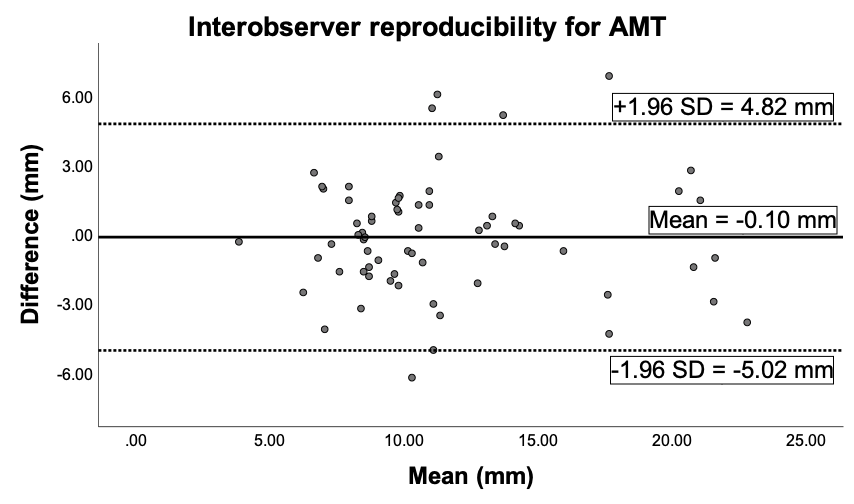

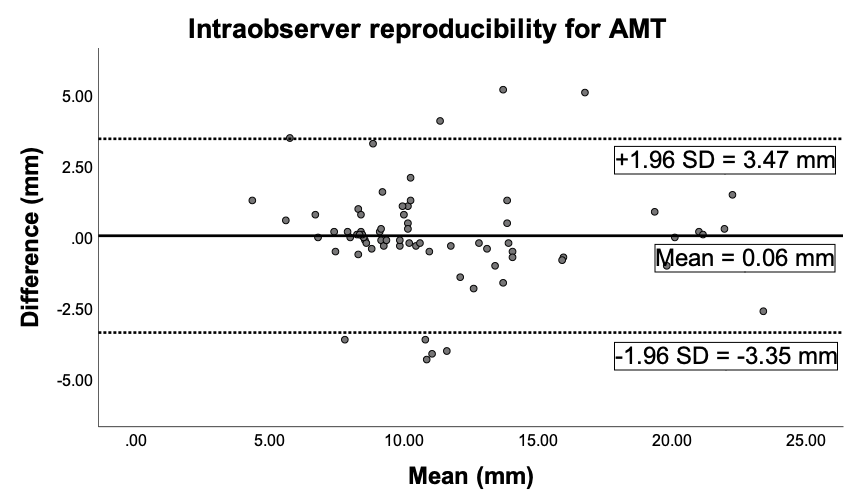

Supplement: Supplementary file 4 — Figure S1 Bland–Altman plots of intraobserver and interobserver reproducibility for each evaluated parameter and each set of images. AMT, adjacent myometrial thickness; CS, Cesarean section; RMT, residual myometrial thickness. [file UOG-60-396-s003.docx]
